# Tincture of Creasote

**Published:** 1842-10-29

**Authors:** 


*Tincture of Creasote.*

| | |
|---|---|
| Take of Creasote - - - - - - | 24 grains. |
| " Alcohol - - - - - - | 4 drachms. |
| " Tincture of Cochineal - - - - | 2 drachms. |
| " Peppermint Oil - - - - - | 12 drops. |

This tincture forms an admirable remedy against the pain of toothache in many instances, if a piece of cotton moistened with it be applied upon the decayed tooth. A few drops of it mixed with water make an excellent lotion for preserving the gums in a healthy condition.—*Med. Chir. Rev.* Oct., 1842.

